# Neuroretinitis Secondary to Toxoplasma Infection in an Adult

**DOI:** 10.1155/crop/8474953

**Published:** 2026-07-29

**Authors:** Shamila Ladak, M. A. Rehman Siddiqui

**Affiliations:** ^1^ Medical College, Aga Khan University Hospital, Karachi, Pakistan, aku.edu; ^2^ Department of Ophthalmology and Visual Sciences, Aga Khan University Hospital, Karachi, Pakistan, aku.edu

**Keywords:** infection, neuroretinitis, stellate maculopathy, toxoplasmosis, vitritis

## Abstract

**Purpose:**

The purpose of this study is to report a case of neuroretinitis in a healthy adult with active primary Toxoplasma infection.

**Methods:**

This study is a case report.

**Results:**

A 27‐year‐old previously healthy woman presented with sudden‐onset blurred vision and a central scotoma in her left eye. Visual acuity in the affected eye was 20/80. Examination revealed dense vitreous exudation, optic disc edema, and macular star formation. OCT demonstrated optic disc elevation, thickening of the retinal nerve fiber layer, and keratitic precipitates along the posterior vitreous interface. A comprehensive laboratory workup was ordered, in which *Toxoplasma gondii* IgM and IgG antibodies were positive. After 6 weeks of treatment with trimethoprim–sulfamethoxazole, significant resolution of the macular star and disc swelling was observed.

**Conclusion:**

Even in the absence of an immunocompromised state or classic risk factors, toxoplasmosis should be considered a differential diagnosis of neuroretinitis. Early diagnosis and treatment are necessary to prevent permanent vision loss.

## 1. Introduction

Neuroretinitis is an inflammatory disorder of the eye, classically manifesting as a triad of unilateral painless vision loss accompanied by optic disc edema and the appearance of stellate macular exudates [1]. Other findings of neuroretinitis include dyschromatopsia, relative afferent pupillary defect (RAPD), and visual field abnormalities [2]. Typically, most cases of neuroretinitis resolve spontaneously over a period of several months with a favorable prognosis, barring a subset of patients who may experience persistent visual compromise or recurrence of the disease [1].

Etiologies of neuroretinitis include infections, inflammatory disorders, autoimmune diseases, and idiopathic causes. The most common infectious cause is cat‐scratch disease, accounting for up to two‐thirds of the cases [3]. Although *Toxoplasma gondii* usually presents as chorioretinitis, marked by focal necrotizing retinitis adjacent to an inactive retinochoroidal scar, one of the unusual manifestations of ocular toxoplasmosis includes neuroretinitis. We report a case of a young patient with unilateral Toxoplasma neuroretinitis.

## 2. Case Presentation

**Figure 1:**
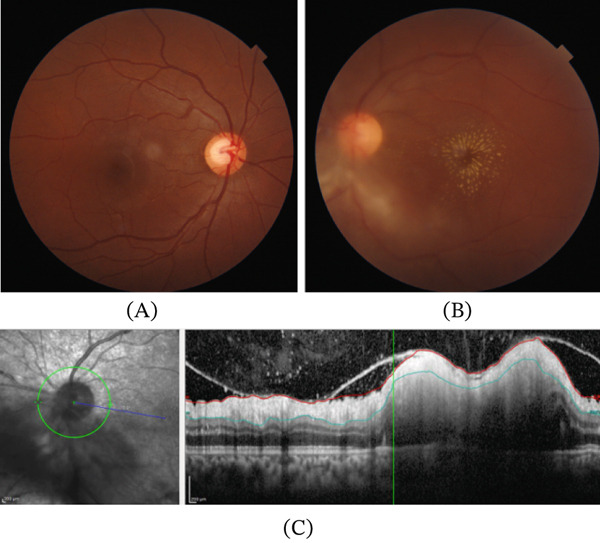
(A) Color fundus photo of the right eye on presentation: no significant findings. (B) Color fundus photo of the left eye on presentation: optic nerve swelling, macular star, and vitreous inflammation. (C) OCT of the left eye on presentation: cellular activity in the vitreous and localized area of full‐thickness retinitis.

A 27‐year‐old previously healthy woman presented to the ophthalmology clinic with a 3‐day history of sudden‐onset blurry vision, photophobia, and a central “black spot” in the left eye. Visual acuity was 20/80 in the left eye and 20/20 in the right eye. The external and anterior segment exams were unremarkable bilaterally. RAPD was negative, but delayed pupillary constriction was observed in the left eye. Intraocular pressure (IOP) measured 12 mm Hg in the right eye and 20 mm Hg in the left eye. Fundus examination of the right eye showed no significant findings (Figure 1A), whereas the left eye showed optic disc edema (2+), dense vitreous exudation, macular star, and an area of active retinitis inferonasal to the star (Figure 1B). Optical coherence tomography (OCT) of the left eye showed an elevated optic disc, pigment epithelial detachment with subretinal fluid, thickening of the retinal nerve fiber layer (RNFL), and keratitic precipitates at the posterior vitreous interface (Figure 1C). There was no evidence of a pre‐existing scar in either eye. Her systemic examination was unremarkable.

The patient reported a 3‐month history of generalized muscle aches and an episode of fever (102 F) 2 weeks before her vision decreased. She denied any history of travel, exposure to pets, tick bites, multiple partners, immunocompromised state, or significant family history for autoimmune conditions. Diagnostic workup included complete blood count (CBC), erythrocyte sedimentation rate (ESR), liver function tests (LFTs), angiotensin‐converting enzyme (ACE), C‐reactive protein (CRP), toxoplasmosis serology, calcium, and 25‐hydroxy vitamin D levels. *Bartonella henselae* serology was unavailable.

**Figure 2:**
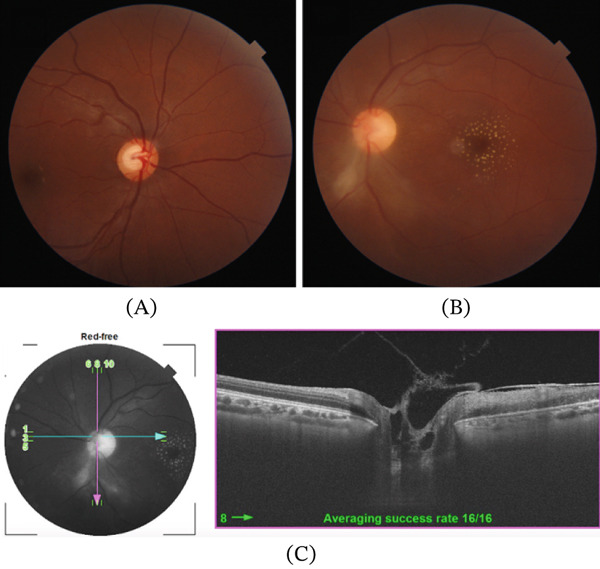
(A) Color fundus photo of the right eye after 6 weeks on TMP‐SMX: no significant findings. (B) Color fundus photo of the left eye after 6 weeks on TMP‐SMX: macular star has partially resolved, and optic nerve swelling and vitritis have improved. (C) OCT of the left eye after 6 weeks on TMP‐SMX: vitritis and retinitis have significantly resolved.

Toxoplasma antibodies IgM and IgG were both positive (> 250 IU/mL), whereas the remaining lab values were within normal limits. The patient was treated with double‐strength trimethoprim–sulfamethoxazole (TMP‐SMX) 160–800 mg twice daily for 6 weeks, in combination with oral prednisolone 20 mg daily and topical nepafenac. At the seventh‐week follow‐up, visual acuity in the left eye had improved to 20/30. No changes were noted in the right eye (Figure 2A). Fundus imaging of the left eye showed resolution of the optic disc edema and vitreous inflammation with partial macular star resolution in the left eye (Figure 2B). OCT confirmed complete resolution of subretinal fluid (Figure 2C).

## 3. Discussion

Neuroretinitis is an atypical presentation of ocular toxoplasmosis. In addition to the classic findings of optic disc edema and macular star formation, anterior chamber (AC) inflammation, macular edema, chorioretinal scarring, and peripapillary vascular sheathing have been reported with Toxoplasma neuroretinitis [4, 5]. Most patients exhibit unilateral or bilateral blurred vision and visual field defects, the most common being a centrocecal scotoma, which was also seen in our patient. Our patient developed neuroretinitis in the absence of classic risk factors or history of exposure to any potential pathogens. However, she reported a 3‐month history of persistent myalgia and a febrile episode preceding her ocular symptoms, fitting into the typical presentation of toxoplasmosis.

Ocular toxoplasmosis is one of the most common causes of posterior uveitis and typically presents as focal necrotizing retinochoroiditis [6]. Multifocal segmental retinal arteritis, vitritis, punctate extraretinal toxoplasmosis, retinal vascular occlusion, secondary Coats′ disease, and sclerochoroiditis have also been reported, although less frequently [6]. Systemically, toxoplasmosis presents with prodromal symptoms resembling mononucleosis, such as fever and cervical lymphadenopathy, and, less commonly, with complications such as polymyositis and myocarditis. Infection in healthy immunocompetent adults can be asymptomatic in about 50% of the cases [7].

A thorough evaluation is essential before classifying neuroretinitis as idiopathic due to the risk of undertreating the patient with only steroids. In cases of toxoplasmosis, corticosteroid use without the administration of antiparasitic drugs may lead to proliferation of the parasite in the eye and cause necrosis. Approximately one‐half of neuroretinitis cases are idiopathic, whereas the remaining are divided into infectious and noninfectious causes. Inflammatory disorders including sarcoidosis, systemic lupus erythematosus (SLE), Behcet′s disease, polyarteritis nodosa, Takayasu arteritis, and inflammatory bowel disease (IBD) account for the majority of noninfectious neuroretinitis [8]. Among infectious causes, *B*. *henselae* (cat scratch disease) remains the most frequently reported etiology [9]. Other bacterial causes of neuroretinitis include *Borrelia burgdorferi*, *Mycobacterium tuberculosis*, *Treponema pallidum*, *Rickettsia rickettsii*, *Leptospira interrogans*, and *Actinomyces israelii* [8–10]. Viral (varicella zoster, herpes simplex, Epstein–Barr, West Nile, Zika, and Chikungunya), fungal (*Coccidioides* and *Histoplasma capsulatum*), and parasitic (*T. gondii* and *Toxocara*) etiologies have also been reported [10, 11]. Serology testing for Toxoplasma IgM and IgG can help distinguish newly acquired from chronic disease. In our case, elevated IgM and IgG titers supported a recent primary infection rather than reactivation, which would have otherwise necessitated further testing.

Toxoplasma neuroretinitis has an overall favorable visual prognosis, with most cases achieving baseline visual acuity when appropriately treated. The most commonly used antibiotic regimens include a combination of pyrimethamine, sulfadiazine, and folinic acid, or TMP‐SMX, with comparable efficacy [12]. TMP‐SMX is relatively well‐tolerated, with milder side effects than the other antibiotic regimens. Oral steroids are used during the active phase to reduce retinal inflammation and further collateral tissue damage, to prevent blood‐retinal barrier breakdown, and to reduce Toxoplasma scarring. Thus, our patient was started on TMP‐SMX and prednisolone, after which she reported significant improvement in her vision.

This case expands on the existing literature on Toxoplasma neuroretinitis and highlights that ocular toxoplasmosis should be considered a potential differential diagnosis for neuroretinitis, even in cases where pertinent risk factors like immunocompromised state are absent. Early detection and serology testing can lead to prompt treatment, enhancing the likelihood of successful resolution and preventing permanent blindness.

## Funding

No funding was received for this manuscript.

## Ethics Statement

This report does not contain any personal identifying information. Verbal informed consent was obtained from the patient for publication of this case report and any accompanying images. The patient was lost to follow‐up before written consent could be obtained.

## Conflicts of Interest

The authors declare that they have no known competing financial interests or personal relationships that could have appeared to influence the work reported in this paper.

## Data Availability

All data and supplementary information are included in this article.
